# Study of the Influence of Sintering Atmosphere and Mechanical Activation on the Synthesis of Bulk Ti_2_AlN MAX Phase Obtained by Spark Plasma Sintering

**DOI:** 10.3390/ma14164574

**Published:** 2021-08-14

**Authors:** Christopher Salvo, Ernesto Chicardi, Cristina García-Garrido, Rosalía Poyato, José A. Jiménez, R. V. Mangalaraja

**Affiliations:** 1 Departamento de Ingeniería Mecánica, Facultad de Ingeniería, Universidad del Bío-Bío, Concepción 4081112, Chile; 2 Departamento de Ingeniería y Ciencia de Materiales y del Transporte, Universidad de Sevilla, 41092 Sevilla, Spain; echicardi@us.es; 3 Instituto Andaluz del Patrimonio Histórico (IAPH), Camino de los Descubrimientos s/n., 41092 Sevilla, Spain; cristina.g.garrido@juntadeandalucia.es; 4 Instituto de Ciencia de Materiales de Sevilla (ICMSE-CSIC), 41092 Sevilla, Spain; rpoyato@us.es; 5 Centro Nacional de Investigaciones Metalúrgicas, Consejo Superior de Investigaciones Científicas (CENIM-CSIC), 28040 Madrid, Spain; jimenez@cenim.csic.es; 6 Laboratorio de Cerámicos Avanzados y Nanotecnología, Departamento de Ingeniería de Materiales, Universidad de Concepción, Concepción 4070386, Chile

**Keywords:** MAX phase, Ti_2_AlN, spark plasma sintering, mechanical activation, milling

## Abstract

The influence of the mechanical activation process and sintering atmosphere on the microstructure and mechanical properties of bulk Ti_2_AlN has been investigated. The mixture of Ti and AlN powders was prepared in a 1:2 molar ratio, and a part of this powder mixture was subjected to a mechanical activation process under an argon atmosphere for 10 h using agate jars and balls as milling media. Then, the sintering and production of the Ti_2_AlN MAX phase were carried out by Spark Plasma Sintering under 30 MPa with vacuum or nitrogen atmospheres and at 1200 °C for 10 min. The crystal structure and microstructure of consolidated samples were characterized by X-ray Diffraction, Scanning Electron Microscopy, and Energy Dispersive X-ray Spectroscopy. The X-ray diffraction patterns were fitted using the Rietveld refinement for phase quantification and determined their most critical microstructural parameters. It was determined that by using nitrogen as a sintering atmosphere, Ti_4_AlN_3_ MAX phase and TiN were increased at the expense of the Ti_2_AlN. In the samples prepared from the activated powders, secondary phases like Ti_5_Si_3_ and Al_2_O_3_ were formed. However, the higher densification level presented in the sample produced by using both nitrogen atmosphere and MAP powder mixture is remarkable. Moreover, the high-purity Ti_2_AlN zone of the MAX-1200 presented a hardness of 4.3 GPa, and the rest of the samples exhibited slightly smaller hardness values (4.1, 4.0, and 4.2 GPa, respectively) which are matched with the higher porosity observed on the SEM images.

## 1. Introduction

The ternary carbides and nitrides with stoichiometry M_n+1_AX_n_ are known as MAX phases, where M is an early transition metal (Ti, Zr, Nb, Cr, Mo, etc.), A is an IIIA and IVA-group element (Al, Si, Sn, Ge, etc.), and X is C or N [1]. The M and X atoms form layers of M_6_X that are interspersed with layers of pure A atoms. The different MAX stoichiometries are classified according to the number of M layers separated by the A layers: M_2_AX (211), M_3_AX_2_ (312), and M_4_AX_3_ (413) [2]. Moreover, it is remarkable that these compounds adopt a hexagonal crystalline structure with *P*6_3_/mmc space group symmetry. These ternary carbides or nitrides combine the properties of ceramics and metals, exhibiting high electrical and thermal conductivities, good thermal shock resistance, superior oxidation resistance, corrosion resistance, and ease to be machined [3]. These combined properties are emanated from their atomic bonding and compacted crystalline structure. Concretely, the M-X atoms form strong directional covalent bonds in the M-X layer, while M-M and M-A bonds are metallic bonds [4].

The MAX phases are potential materials thanks to those exciting properties to be implemented in advanced applications under severe conditions, such as high temperature, aggressive corrosion, and high irradiation in aerospace or nuclear systems [5,6]. Concretely, the MAX phases based on the Ti-Al-N and Ti-Al-C ternary systems combine lightweight and corrosion-resistant properties, making them attractive for industrialization [7]. All M_2_AX phases exhibit good thermal and electrical conductivities, high hardness, and machinability characteristics of metals alongside oxidation resistance, damage tolerance, and thermal stability even at temperatures as high as 1400 °C [8]. However, the complete conversion of the raw powder materials to the Ti_2_AlN is difficult due to the coexistence of various phases in the different temperatures at which the Ti_2_AlN is stable [9,10]. There is still a lack of knowledge about the effect of sintering parameters to obtain a single bulk Ti_2_AlN MAX phase.

For the production of the Ti_2_AlN MAX phase, several powder metallurgy processes have been used, such as self-propagating high-temperature synthesis (SHS) [11,12,13], microwave sintering [14], hot pressing (HP) [15,16,17,18,19,20], hot isostatic pressing (HIP) [13,21,22], and spark plasma sintering (SPS) [23,24,25,26,27,28,29]. The processing approach may be used depending on starting powder mixtures such as Ti+Al (milled in a nitrogen atmosphere), Ti+AlN, TiN+AlN, Ti+TiN+AlN, Ti+Al+TiN, under vacuum, argon, or nitrogen. In this context, Kovalev et al. [30] noticed the dissociation and evaporation of AlN at high temperatures processing, which inhibited the synthesis of a Ti_2_AlN single phase. Chlubny et al. [13] stated that the use of nitrogen pressure on SHS promoted the formation of a mixture of different MAX phases [13]. Nam et al. [31] reported an increment of the Ti_2_AlN amount and its lamellar thickness by increasing the nitrogen atmosphere levels on Ti alloys. Moreover, it has been suggested that diluting the samples with metal nitrides may enhance the degree of conversion [11]. On the other hand, the use of mechanically activated powders (MAP) has been considered for a practical approach to diminish the time and temperature required to obtain the Ti_2_AlN due to the incremented reactivity of the as-milled powders associated with the reduced segregation of constituents, the smaller sized powders, and its rougher surfaces, with the consequent increase of the reactive surface [30,32].

The SPS has demonstrated the economic and technological advantages of processing and forming single phases in shorter soaking time and lower sintering temperatures among the processing approaches mentioned. The SPS is a field-assisted sintering technique (FAST) in which a uniaxial compaction pressure is applied to the sample along with a pulse direct current to obtain the fully dense and nearly single phase of MAX phases [33,34,35,36]. It is reported that by processing a powder mixture of Ti and AlN by SPS, a solid-state reaction between them will generate the Ti_2_AlN MAX phase under several associated steps. The mechanism of formation and the reaction sequences between 800 and 1450 °C that occur during synthesis by reactive SPS of Ti and AlN powder mixtures have been previously discussed. In previous work, the lower sintering temperature (1200 °C) at which we nearly achieved a single-phase bulk Ti_2_AlN MAX (>98% purity) starting from the Ti/AlN powder mixture and processed through reactive SPS was reported [28].

Therefore, this work studied the effect of using a mechanical activation process and nitrogen atmosphere on the synthesis, microstructure, and mechanical properties of Ti_2_AlN MAX phase bulk materials obtained by SPS. For this goal, stoichiometric mixtures of Ti and AlN powders were prepared. A high-energy milling process was carried out to mechanically activate a part of these powders under an argon atmosphere. Both unmilled and milled powders were consolidated at the same temperature by SPS at 1200 °C under 30 MPa with vacuum or nitrogen atmospheres for 10 min. A detailed structural and microstructural characterization were performed to determine the feasibility of the milling and the consolidation process to obtain bulk Ti_2_AlN MAX phase composites.

## 2. Materials and Methods

### 2.1. Mixing and Characterization of As-Milled Powders

Aluminum nitride, AlN (325 mesh, CAS number 24304-00-5, O: 7500 ppm, C: 300 ppm, and N: 33.47%), and pure titanium, Ti (99.5% purity, -325 mesh, CAS number 7440-32-6, O: 0.22%, C: 0.01% and N: 0.01%), from Alfa Aesar Chemicals company (Tewksbury, MA, USA) were used as raw materials in this work to synthesize Ti_2_AlN MAX phase.

Concretely, 20 g of mixtures of Ti and AlN starting powders were prepared in a molar ratio of 2:1 by mixing in a Turbula^®^ T2F mixer for 4 h. A set of these mixtures were milled for 10 h under argon (99.999% purity, Indura, Chile) using a Retsch^®^ PM400 planetary mill (Haan, Germany) at 200 rpm and adding a one wt.% of stearic acid from Sigma-Aldrich (CAS number 57-11-4, 99.5% purity, Michigan, USA) as a process control agent (PCA) to avoid the excessive welding and agglomeration of powders, which helps to reduce the particle size. An agate vessel of 250 mL and balls of 9 mm of diameters were used, resulting in a ball to powder weight ratio (BPR) of 10:1. An on-off cycle of 20–10 min was programmed to avoid the overheating of the vial and maintain the room temperature to remove the local increase of temperature in the synthesis process. Subsequently, the morphology and particle size of the powder mixtures were determined by scanning electron microscopy (SEM), JEOL model JSM-6380LV. The X-ray diffraction (XRD) measurements were carried out to characterize the crystalline phases present in the samples by using a Bruker AXS D4 Endeavor diffractometer with Cu-K_α_ radiation (Bruker AXS, Karlsruhe, Germany). The XRD data were recorded in the conventional Bragg–Brentano geometry for 2θ scans ranging from 30 to 90° with a step width of 0.02° and a counting time of 2 s/step. A current of 20 mA and a voltage of 40 kV were employed as tube settings. The crystalline phases present in the XRD patterns were determined using the DIFFRACplus EVA software by Bruker AXS and the Powder Diffraction Files (PDF) database from the International Centre for Diffraction Data (ICDD). The microstructural information and phase quantification were performed by fitting the whole measured diffraction pattern with version 4.2 of the TOPAS (Bruker AXS) Rietveld analysis software. The crystallographic information was obtained from Pearson’s crystal structure database.

### 2.2. Reactive Sintering of Powders

The reactive sintering of the as-mixed (NMAP) and as-milled (MAP) powders was carried out by the spark plasma sintering (SPS) technique. The powder mixtures were introduced in a graphite die with an inner diameter of 15 mm and placed into the spark plasma sintering device (Model 515S, SPS Sinter Inc., Kanagawa, Japan). The reactive sintering processes were carried out with a heating rate of 100 °C/min up to the sintering temperature of 1200 °C, 10 min of dwell time, and 30 MPa of uniaxial pressure. Both nitrogen and vacuum atmospheres (10 Pa) were used for comparison purposes regarding the sintering atmosphere. The temperature evolution during the sintering process was measured with an optical pyrometer by focusing on the side of the graphite die. The reactive sintered specimens were denoted as MAX-1200, MAX-1200M, MAX-1200N, and MAX-1200NM, where N means nitrogen atmosphere and M uses mechanically activated powders.

### 2.3. Characterization of the Reactive Sintered Specimens

The X-ray diffraction (XRD) measurements were carried out similarly to those carried out on the powders as described in Section 2.1. The microstructural information and phase quantification were performed by fitting the whole measured diffraction pattern with version 4.2 of the TOPAS (Bruker AXS) Rietveld analysis software. The absolute density of the samples was determined by the Archimedes method according to the ASTM B962-17 standard. The theoretical densities of the pieces were obtained by using the rule of mixture, considering the phase content obtained by the Rietveld refinement. The mechanical properties at room temperature of consolidated materials were evaluated using a microhardness tester, supplied by Struers, and by an average value taken from the five indentations carried out by applying a load of 10 N on a well-polished surface during 10 s at different locations on different fully densified zones of each sample. The microstructure, morphology and particle size, and composition of different phases for the reactive sintered specimens were analyzed on both polished and fractured surfaces of the sintered samples using a HITACHI field emission scanning electron microscope (FESEM), model S-4800, equipped with a Bruker-XFlash 4010 energy dispersive X-ray spectrometer (EDS) and a 5 kV of acceleration voltage for SEM images, and 20 kV for EDS determination. Finally, to clarify and corroborate the phases formed during the reactive sintering process and the distribution of these phases due to their possible segregations, X-Ray MicroDiffractions (XRMD) were collected in different areas specimens using a D8 DISCOVER A25 diffractometer (Bruker, MA, USA).

## 3. Results and Discussion

**Figure 1:**
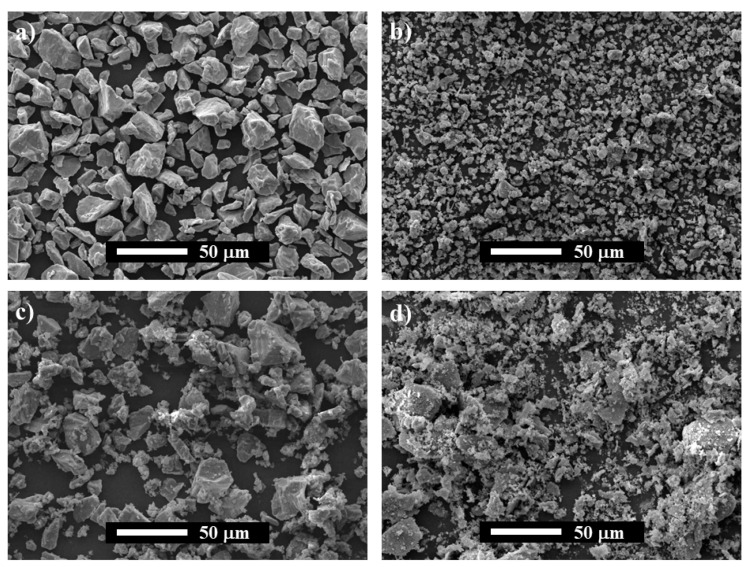

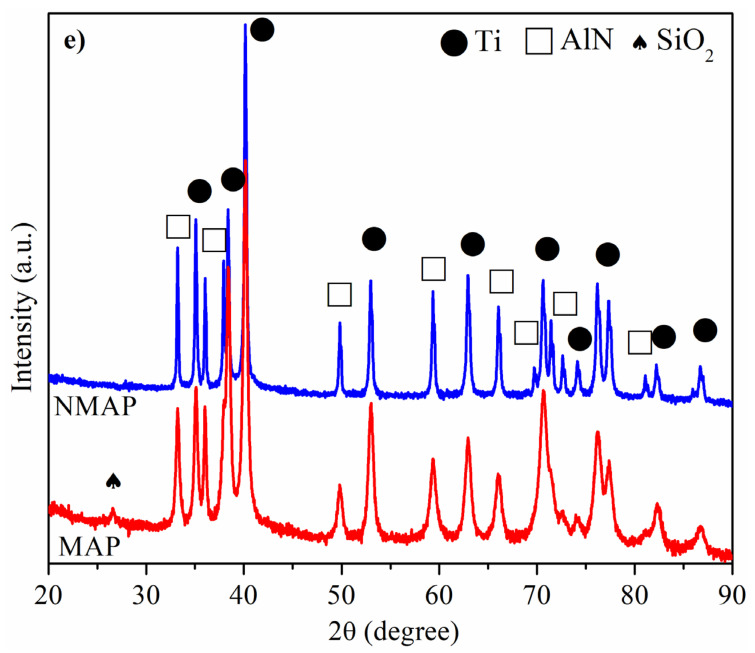
SEM images of powders: (**a**) as-received Ti, (**b**) as-received AlN, (**c**) Non-Mechanically Activated Powder mixtures (NMAP) and, (**d**) Mechanically Activated Powder mixtures (MAP); (**e**) XRD of NMAP and MAP powder mixtures.

**Figure 2:**
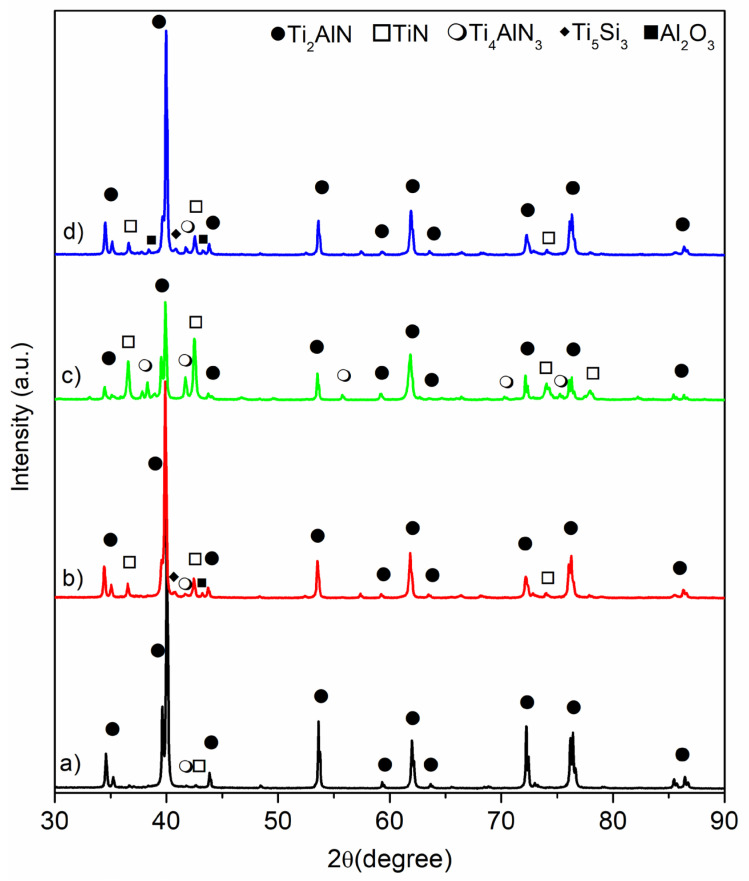
XRD patterns of the bulk Ti_2_AlN MAX phase specimens: (**a**) MAX-1200, (**b**) MAX-1200M, (**c**) MAX-1200N, and (**d**) MAX-1200NM.

The morphology of the as-raw powders (Ti and AlN) and NMAP and MAP powder mixtures were studied by SEM and shown in Figure 1. As can be observed, the starting Ti and AlN have shown angular and equiaxed morphology with an average mean size lower than 20 and 5 µm, respectively. On the other hand, it is noticeable that, during the milling process, the particles of Ti are plastically deformed, and the AlN particles exhibited a progressive decrease in their average particle size. Thus, the MAP powder mixture shows plate-like large Ti particles with tiny particles of AlN agglomerated to their surfaces. The microstructural evolution of the powders was studied by comparing their XRD patterns, which mainly consist of a broadening of the Ti and AlN diffraction peaks. Only an additional peak of SiO_2_ (JCPDS card No.: 46-1045) is evidenced on the MAP sample, related to contamination from the agathe jars and balls. As no additional peaks are observed in Figure 2b, and the peak positions of Ti and AlN did not shift, it is concluded that both phases did not react during the mechanical activation process.

The X-ray diffraction (XRD) patterns of samples sintered by SPS at 1200 °C in vacuum (MAX-1200 °C), in nitrogen (MAX-1200N), mechanically activated and sintered in vacuum (MAX-1200M), and mechanically activated and sintering in nitrogen (MAX-1200NM) are shown in Figure 2. The phases present in these diffraction patterns are identified by the search-match technique using the database from the International Centre for Diffraction Data (ICDD). For the MAX-1200 (Figure 2a), the main phases presented are Ti_2_AlN (ICDD card No.: 18-0070), TiN (ICDD card No.: 38-1420), and Ti_4_AlN_3_ MAX phase (ICDD card No.: 65-9771). On the other hand, for the MAX-1200M y MAX-1200NM (Figure 2b,d respectively), the phases indexed are similar, i.e., Ti_2_AlN, Ti_4_AlN_3,_ and TiN. However, other minor phases, due to their slight peaks, were indexed. Concretely, they are Ti_5_Si_3_ (ICDD card No.: 29-1362) and Al_2_O_3_ (ICDD card No.: 46-1212). It is important to note that the presence of both the last new phases indexed, Ti_5_Si_3_ and Al_2_O_3_, in contrast with the non-mechanically activated specimen (MAX-1200), are a direct consequence of the milling process. Concretely, the Si and O to form the Ti_5_Si_3_ and Al_2_O_3_, respectively, come from the agate milling media released during milling and incorporated into the powder mixtures previously to SPS. Thus, during SPS, the SiO_2_ reacts with raw materials to form both the abovementioned non-desirable phases.

On the other hand, for the MAX-1200N (Figure 2c), obtained by using a nitrogen atmosphere, the phases presented are Ti_2_AlN, TiN, Ti_4_AlN_3,_ AlN (ICDD card No.: 08-0262), and Ti_3_Al (ICDD card No.: 14-0451). Most of these are nitrogenous phases due to the N_2_ atmosphere that reacts with the powder mixtures during SPS. Several reactions have been proposed and can take place for the reaction between Ti and AlN raw materials and other Ti and Al intermediate reaction phases [23,25]:

Ti + AlN → Ti(Al,N)(1)

Ti + AlN → TiN + Al(2)

Ti + Al → TiAl(3)

3Ti + Al → Ti_3_Al (4)

Ti_3_Al + N → Ti_3_AlN (5)

Ti_3_Al + 2Al → 3TiAl (6)

2Ti_3_Al + Al + N → 3Ti_2_AlN (7)

2Ti + AlN → TiAl + TiN(8)

TiN + TiAl → Ti_2_AlN (9)

The mentioned reactions do not necessarily follow the above order. In general, when the Al and N atoms of AlN presented enough energy to diffuse across the interface (above 800 °C), they entered into the defects of the Ti phase, mainly reacting to form a Ti (Al,N) solid solution and TiN (Equations (1) and (2)) [37]. Due to this, the amount of Al changed gradually in the reaction layer, and by increasing the temperature, several Ti-Al and Ti-Al-N intermediate phases were formed (Equations (3)–(8)) [25]. It has been reported that the Al atoms are substitutionally dissolved on the Ti atomic positions, obtaining a wide variety of Ti-Al intermetallics such as TiAl, Ti_3_Al, Al_3_Ti, Al_2_Ti [6]. The experimental data evidence that the formation of Ti_2_AlN concludes with the reaction between TiN and TiAl (Equation (9)) [18,32].

The quantitative phase analysis was carried out to evaluate the use of mechanically activated powders and different sintering atmospheres (vacuum or N_2_) on the efficiency of the synthesis route to obtain single Ti_2_AlN MAX materials. The results of quantitative Rietveld analysis using the room-temperature crystal structure information of the phases previously identified in the qualitative research are shown in Table 1. The GoF values were lower than 2, which suggests good refinements. However, the presence of other crystalline phases presented under the XRD quantification sensibility cannot be excluded. It is noticeable that the MAX-1200 is the sample that gave the higher Ti_2_AlN MAX phase percentage (98 wt.%), and further information about its synthesis and characterization has been reported previously by focusing on the sintering temperature effects [28]. Some researchers once said that the Ti_2_AlN MAX phase obtained by the SPS method also turned out to be accompanied by secondary undesired phases, reaching up to 10 wt.%, which depended mainly on the consolidation parameters and starting powders [25,26,38]. In this work, the consolidation by SPS of a MAP powder mixture of Ti and AlN (MAX-1200M) reduced the quantity of Ti_2_AlN MAX phase up to 82 wt.% due to the formation of secondary phases such as TiN (9 wt.%), Al_2_O_3_ (5 wt.%), Ti_5_Si_3_ (<2 wt.%) and Ti_4_AlN_3_ (<2 wt.%). The appearance of the TiN is associated with using the MAP mixture, one of the finest and consequently better distributed AlN powders, accelerating the diffusion rate of the Al and N atoms during the sintering process, which generates a local depletion with aluminum atoms favoring the TiN formation. Similar phases were reported during the synthesis of Ti_2_AlN by HP of a MAP powder mixture of Ti and AlN [32], which formed the Ti_4_AlN_3_ MAX phase in SPS. This phase corresponds to the 413 MAX phase, and studies on it are scarce [38,39]. Previously, the formation of the Ti_4_AlN_3_ on the Ti and AlN powder sintering by SPS has been attributed to the conditions that occurred only at the border of the sample [28]. Other previous studies explained the formation of the Ti_4_AlN_3_ based on a transformation from a MAX phase of a low *n* order to a higher-order [40]. The use of nitrogen gas as a sintering atmosphere (MAX-1200N) seems to be the worst SPS sintering condition. It induced the excessive formation of undesirable nitrogenous phases, such as the TiN and Ti_4_AlN_3_, reaching amounts of 41 wt.% and 17 wt.%, respectively. According to the previously stated [13], the use of nitrogen pressure on SHS promotes the formation of different MAX phases. Moreover, it is noticeable that by using MAP powders at nitrogen atmosphere (MAX-1200NM), the phase amounts of the MAX-1200NM are similar to those obtained for the MAX-1200M, and the high purity of the Ti_2_AlN is retained. This behavior suggests that the formation of TiN and Ti_4_AlN_3_ in MAX-1200N occurs during the formation of Ti_2_AlN due to the excessive presence of N. The crystallite size of the Ti_2_AlN is similar in the MAX-1200 and MAX-1200N and slightly lower for the MAX-1200M and MAX-1200NM. These are associated with secondary phases such as Ti_5_Si_3_ and Al_2_O_3_, which may be obstacles to the coarsening of the crystallites. The crystallite size determined from XRD patterns provides the size of the coherent scattering domains, which correspond to regions with perfect crystalline order. On the other hand, it is not possible to conclude what the effect of nitrogen gas on the crystallite size of the nitrogenous phases is because its size does not present a clear trend. However, the crystallite size of the Ti_4_AlN_3_ is larger for all the conditions, although mainly when nitrogen gas and the mechanical activation process (MAX-1200NM) were used. 

**Figure 3:**
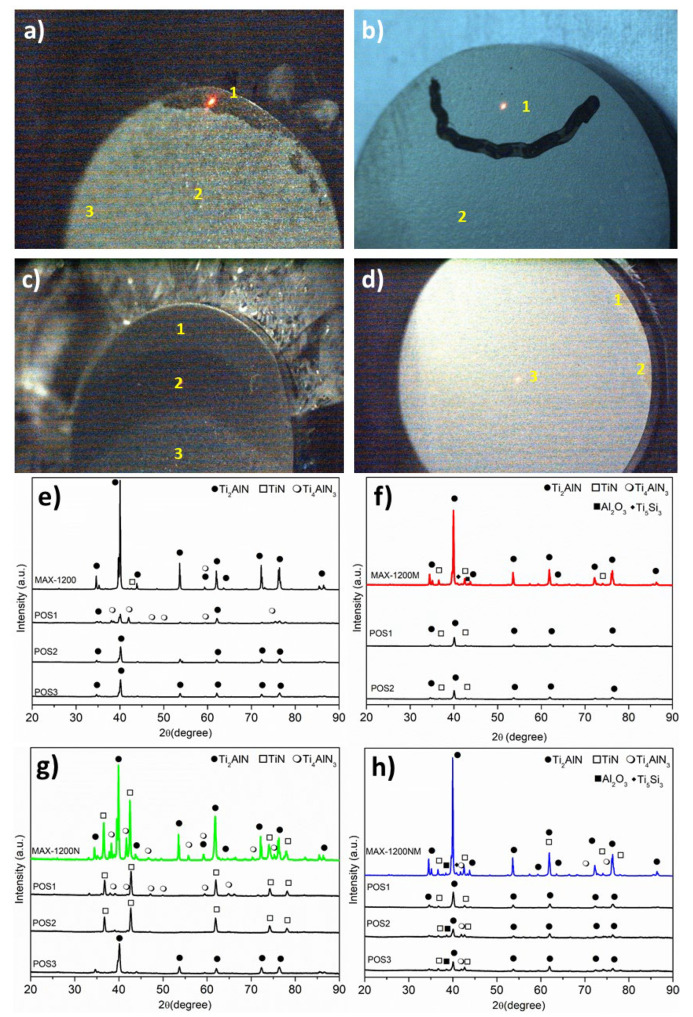
Macroscopic view of the samples and their micro-XRD results for: (**a**,**e**) MAX-1200, (**b**,**f**) MAX-1200M, (**c**,**g**) MAX-1200N, and (**d**,**h**) MAX-1200NM.

A visual inspection of all samples (Figure 3) evidenced that the pieces presented a homogeneous microstructure in general, except the MAX-1200N, which clearly shows a heterogeneous microstructure (Figure 3c). For a better understanding, several micro-XRD analyses on different zones of the samples were performed and are marked with yellow numbers above the corresponding image. For the case of the MAX-1200, the presence of the Ti_4_AlN_3_ (Position no.1, Figure 3e) is confirmed, which is formed on the border of the sample, as we previously reported [28]. The formation of Ti_2_AlN by SPS of Ti and AlN powders has been previously described [25]. Concretely, at high temperatures, TiN and Ti-Al compounds are formed together with various Ti-Al phases such as TiAl, Ti_3_Al, Al_3_Ti, and Al_2_Ti. Finally, due to the solubility of the N atoms, TiN and TiAl react and precipitate as Ti_2_AlN [17,19]. The three zones of the MAX-1200N were studied, and the micro-XRD patterns of POS1, POS2, and POS3 are shown in Figure 3f, evidencing that the zones 1, 2, and 3 corresponded to Ti_4_AlN_3_/TiN, TiN, and Ti_2_AlN, respectively. On the other hand, the MAX-1200M and MAX-1200NM showed a homogeneous microstructure, presenting similar phases at the different zones studied, and the MAX-1200NM showed some Ti_4_AlN_3_ rich zones on the border. Then, it is concluded that the use of MAP powders for the nitrogen atmosphere retained the Ti_2_AlN MAX phase microstructure at the macroscopic level.

**Figure 4:**
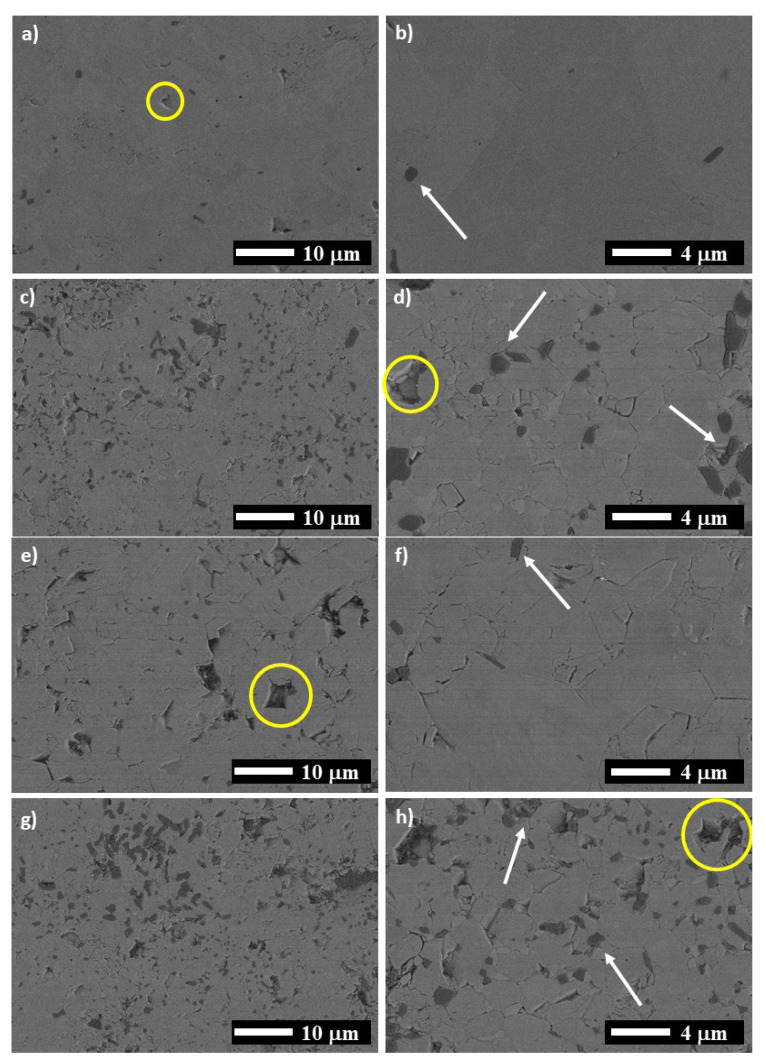
SEM images for: (**a**,**b**) MAX-1200, (**c**,**d**) MAX-1200M, (**e**,**f**) MAX-1200N and (**g**,**h**) MAX-1200NM.

Several SEM images of the samples taken in the zones of the high purity Ti_2_AlN are presented in Figure 4. For the MAX-1200, elongated grains of Ti_2_AlN with tiny particles of Al_2_O_3_ presented on the grain boundaries are shown (Figure 4a,b). The particles of Al_2_O_3_ are formed due to the reaction at high temperatures of the Al, with some atoms of O incorporated into the starting powders as contamination. For all samples, the Al_2_O_3_ particles are marked with white arrows and the porosity with yellow circles. Barsoum et al. [21] also reported the formation of Al_2_O_3_ during the consolidation of Ti_2_AlN by HIP, in which the only source of oxygen was the raw powders used (1.27 wt.%).

On the other hand, the MAX obtained using MAP powders at vacuum (Figure 4c,d), MAX-1200M) presented a smaller grain size for the Ti_2_AlN, a higher quantity of Al_2_O_3_ particles, and pores of around 2 μm in size. The MAX-1200N has appreciated pores of 5 to 6 μm in size and grains slightly larger than those observed for MAX-1200M (Figure 4e,f) Finally, the MAX-1200NM exhibited a high presence of Al_2_O_3_ particles and a similar grain size to the MAX-1200M. In general, the higher amount of Al_2_O_3_ in the samples produced by using MAP powder mixtures is associated with the decomposition of the SiO_2_ particles at high temperatures, which had been added to the starting powder as contamination during the mechanical activation process.

The characteristics of the bulk Ti_2_AlN compounds obtained by spark plasma sintering in this work are presented in Table 2. The theoretical densities of the samples were calculated according to the rule of mixtures, using the density values of 4.3, 4.7, 5.4, 3.6, 4.3, 3.9, and 3.4 g/cm^3^ for Ti_2_AlN, Ti_4_AlN_3_, TiN, AlN, Ti_5_Si_3_, Al_2_O_3,_ and TiAl_3_, respectively. The volume content of each phase was calculated using the wt.% obtained by the Rietveld refinements presented in Table 1. It is essential to mention that the MAX-1200 exhibited the highest densification of all samples, reaching ~97% of relative density. The relative density for the other samples such as MAX-1200M, MAX-1200N, and MAX-1200NM is ~95, ~92, and 96%, respectively. These density values matched with the pore quantity as seen in SEM images (Figure 4). According to the literature, reaching full densification in the Ti_2_AlN MAX phase is difficult, even using HIP for 48 h at 1400 °C [21]. It is noticeable that the higher densification level presented in the sample produced by using both nitrogen atmosphere and MAP powder mixture (MAX-1200NM) compared to the MAX-1200M and MAX-1200N samples. The MAP powders are finer and promoted the diffusion of Al and N atoms faster than in the powders not activated, giving more time to the pieces for the densification. The density values are not clearly reported, and the few published reports only mentioned that the samples obtained are nearly fully dense. Cui et al. and Ming et al. reported densification for the Ti_2_AlN of 99 and 97.9%, respectively [23,24]. On the other hand, the high-purity Ti_2_AlN zone of the MAX-1200 presented a hardness of 4.3 GPa, which is in the range of values previously reported for the Ti_2_AlN MAX phase (3.5–4.3 GPa) [17,20,21]. The high-purity Ti_2_AlN zone of the rest of the samples (MAX-1200M, MAX-1200N, and MAX-1200NM) exhibited slightly smaller hardness values (4.1, 4.0, and 4.2, respectively). This is matched with the higher porosity observed on the SEM images (Figure 4).

## 4. Conclusions

Ti_2_AlN MAX phase was successfully prepared from milled and unmilled Ti:AlN powder mixture in a molar ratio of 2:1, respectively, by reactive spark plasma sintering at 1200 °C. The nearly single bulk Ti_2_AlN MAX phase (>98% purity) was successfully prepared from the Ti:AlN powder mixture in a molar ratio of 2:1, respectively, by the reactive spark plasma sintering at 1200 °C in the vacuum condition (MAX-1200). The qualitative analysis of the diffraction peaks of the Ti_2_AlN MAX specimens evidenced that the use of nitrogen atmosphere induced an important change in the phase content of the samples, which exhibited a decrease of the Ti_2_AlN MAX phase content (MAX-1200N). The consolidation of a mechanically alloyed powder mixture of Ti: AlN reduced the quantity of Ti_2_AlN MAX phase up to 82 wt.% due to the formation of phases such as TiN, Ti_5_Si_3,_ and Al_2_O_3_, which were, in turn, due to the presence of SiO_2_ particles introduced as contamination during the mechanical activation process. The use of nitrogen as a reactive sintering atmosphere did not favor the spark plasma sintering condition of the powder mixture of Ti and AlN. An excessive formation of the secondary TiN and Ti_4_AlN_3_ phases was presented, which reached the amounts of 41 and 17 wt.%, respectively. However, the higher densification level presented in the sample was produced by using both nitrogen atmosphere and MAP powder mixture (MAX-1200NM), compared to the MAX-1200M and MAX-1200N samples. Moreover, the high-purity Ti_2_AlN zone of the MAX-1200 presented a hardness of 4.3 GPa, and the rest of the samples (MAX-1200M, MAX-1200N, and MAX-1200NM) exhibited slightly smaller hardness values (4.1, 4.0, and 4.2, respectively) which matched with the higher porosity observed on the SEM images.

## Figures and Tables

**Table 1:** Quantitative analysis of phases obtained by the Rietveld and the quality refinement parameters. GOF: goodness of fit; D: crystallite size.

Sample	Parameters				
	R_exp_	GOF	Phase	wt.%	D (nm)
MAX-1200	7.6	2.3	Ti_2_AlN	>98	156
			TiN	<1	–
			Ti_4_AlN_3_	<1	39
MAX-1200M	7.9	1.7	Ti_2_AlN	82	102
			TiN	9	95
			Al_2_O_3_	5	148
			Ti_5_Si_3_	<2	–
			Ti_4_AlN_3_	<2	77
MAX-1200N	7.9	1.8	Ti_2_AlN	38	166
			TiN	41	60
			AlN	2	68
			TiAl_3_	<2	32
			Ti_4_AlN_3_	17	78
MAX-1200NM	7.9	1.7	Ti_2_AlN	80	116
			TiN	8	113
			Al_2_O_3_	5	196
			Ti_5_Si_3_	3	32
			Ti_4_AlN_3_	4	109

**Table 2:** Characteristics of the Ti_2_AlN MAX phase obtained in this work under the different SPS sintering conditions.

Features	MAX-1200	MAX-1200M	MAX-1200N	MAX-1200NM
Theoretical density (g/cm^3^)	4.31	4.37	4.72	4.36
Measured density (g/cm^3^)	4.18	4.14	4.32	4.19
Relative density (%)	96.9	94.7	91.5	96.1
Hardness (GPa)	4.3	4.1	4.0	4.2

## Data Availability

The data presented in this study are available on request from the corresponding author.
